# Artificial intelligence in cataract grading system: a LOCS III-based hybrid model achieving high-precision classification

**DOI:** 10.3389/fcell.2025.1669696

**Published:** 2025-09-09

**Authors:** Gege Tang, Jie Zhang, Yingqi Du, Dexun Jiang, Yanhua Qi, Nan Zhou

**Affiliations:** ^1^ Department of Ophthalmology, The Second Affiliated Hospital, Harbin Medical University, Harbin, China; ^2^ National Key Laboratory of Laser Spatial Information, Harbin Institute of Technology, Harbin, China; ^3^ Harbin University, Harbin, China

**Keywords:** cataract, neural network, artificial intelligence, anterior segment image, Lens Opacities Classification System III (LOCS III)

## Abstract

**Purpose:**

To design an artificial intelligence (AI) algorithm based on the Lens Opacities Classification System III (LOCS III) to realize automatic diagnosis of cataracts and classification of its.

**Methods:**

This retrospective study develops an AI-based neural network to diagnose cataracts and grade lens opacity. According to the LOCS III, cataracts are classified into Nuclear Opalescence (NO), Nuclear Color (NC), Cortical(C) and Posterior subcapsular(P). The newly developed neural network system uses grayscale, binarization, cluster analysis, “dilation-corrosion” and other methods to process and analyze the images, then the study need to test and evaluate the generalization ability of the system.

**Results:**

The new neural network system can identify 100% of lens anatomy. It has an accuracy of 92.28%–100% in the diagnosis of nuclear cataract, cortical cataract and posterior subcapsular cataract. The classification accuracy rate of the system for cataract NO, NC, C, P is between 90.88% and 100%, the Area Under the Curve (AUC) is between 96.68% and 100%.

**Conclusion:**

A novel cataract diagnostic and grading system can be developed based on the AI recognition algorithm, which establishes an automatic cataract diagnosis and grading scheme. The system facilitates rapid and accurate cataract diagnosis and grading.

## 1 Introduction

Cataract is a main cause of visual impairment and blindness worldwide (Lee and Afshari, 2023). Surgery is the most effective treatment for cataract. Severe cataract can cause complications such as lens nucleus dislocation and glaucoma (Guan et al., 2022) significantly increasing surgical risks. Thus, early and precise diagnosis is clinically critical. In clinical practice, cataract is typically diagnosed under a slit lamp (Brown et al., 1987) and graded using the lens opacity grading system II or III (Chylack et al., 1989; Chylack et al., 1993). However, the accurate diagnosis and grading of lens diseases depend on ophthalmologists’ clinical experience. Different ophthalmologists may evaluate the patient’s eye condition differently based on their years of experience (Lu et al., 2025). In remote areas, limited access to professional ophthalmologists and ophthalmic equipment, along with inconvenient medical conditions, lead to delayed diagnosis and treatment for patients (Mundy et al., 2016).

Artificial intelligence (AI), as an interdisciplinary technological domain, is dedicated to developing computational systems that emulate human cognitive processes (Liu et al., 2021). Its medical applications predominantly utilize machine learning (ML) and deep learning (DL) frameworks for imaging diagnostics (Castiglioni et al., 2021). Ophthalmology currently represents one of the most dynamic frontiers in AI research (Yang et al., 2023), where image-based diagnostic systems show remarkable suitability for traditional ML and DL implementations (Wang et al., 2024). Owing to its exceptional capability in extracting high-level features and latent patterns from massive datasets, DL systems now match clinicians’ performance levels in feature-based diagnostic tasks (Yu et al., 2018). The scope of applications has expanded significantly, progressing beyond its initial focus on diagnosing retinal pathologies (e.g., diabetic retinopathy, age-related macular degeneration, and retinopathy of prematurity) (Oganov et al., 2023; Zhang et al., 2023; Xu et al., 2024) to now include screening for anterior segment conditions such as glaucoma, cataracts, iris abnormalities, and corneal diseases (Ting et al., 2021; Wu et al., 2022). Currently, machine learning and image processing technology are widely used by researchers in their studies to develop cataract detection methods (Wan Zaki et al., 2022; Gali et al., 2019). Many researchers employ various deep learning algorithms (Xu et al., 2020; Wu et al., 2019), such as Convolutional Neural Networks (CNN), Residual Neural Network (ResNet) and Support Vector Machine (SVM) (Imran et al., 2020), to diagnose image-based categorization of cataract as non-cataract, mild, moderate, and severe. However, there is a lack of utilizing deep learning algorithms for simultaneous diagnosis and grading of various types of cataracts in anterior segment images, including nuclear (N) (Li et al., 2010; Li et al., 2009), cortical (C) (Lu et al., 2022), and posterior subcapsular (P) cataract, based on the Lens Opacities Classification System III (LOCS III).

Building an AI model involves several steps, including system data preparation (image preprocessing), dataset partitioning, model construction, optimization, and evaluation (Shao et al., 2023). Prior to implementing algorithms, many researchers perform preprocessing on images to eliminate noise, thereby enhancing the accuracy of feature extraction (Xu et al., 2020). Due to reflection of eyes and local uneven illumination, the quality of the original images is affected. That effect may decrease the accuracy of feature extraction, and consequently impact the reliability of cataract diagnosis and grading. Xu et al. converted the original images from RGB color mode (RGB) color space to the green component images to eliminate the uneven illumination (Xu et al., 2020; Linglin et al., 2017). But they did not preprocess specific areas of the original images indetail. Gan et al. proposed two artificial intelligence diagnostic platforms for cortical cataract classification, dividing the cataract into four stages: incipient stage, intumescent stage, mature stage, and hyper-mature stage (Gan et al., 2023). The platforms did not consider the influence of bright spots in the images and did not provide more detailed classification of cortical cataract. In addition, preprocessing encompasses extracting regions of interest to mitigate the influence of surrounding redundant information. In 1997, researchers proposed a method based on deep learning algorithms to classify the severity of nuclear cataract, which extracted second-order gray-level statistics from within circular regions of the nucleus as image features (Duncan et al., 1997). However, the algorithm did not consider the information of elliptical lens regions, resulting in incomplete extraction of feature information. Li et al. (Li et al., 2010) investigated an algorithm for the automatic diagnosis of nuclear cataract based on the LOCS III that can automatically detect the nucleus region from slit-lamp images using the modified active shape model (ASM) method (Li and Chutatape, 2003), which is critical for assessing nuclear cataract. This article presents an automated nuclear cataract severity classification algorithm that utilizes the YOLOv3 algorithm to locate the nuclear region of the ocular lens (Hu et al., 2020). But, the complexity and large computational burden of YOLOv3 make it challenging to implement.

Currently, no algorithm exists that can comprehensively diagnose and grade all types of cataracts based on LOCS III during initial screening, and the aforementioned article also lacks detailed description of methods for accurate localization of the lens (Litjens et al., 2017; Li et al., 2021; Yousefi et al., 2020). Additionally, existing research has not adequately addressed the impact of extremely bright spots in images on the extraction of ocular features, which may lead to inaccurate diagnostic results. In response to the above issues, the primary contributions of this paper are as follows.

1. Based on deep learning, this study proposes a systematic algorithm that accurately classifies and grades various types of cataracts according to the Lens Opacities Classification System III (LOCS III).
2. The article presents a lens localization algorithm of nuclear type image. The algorithm is based on expanded ellipse traversal that can enhance the accuracy of interested region localization, which can contribute to improved feature extraction precision of Nuclear Cataract images.
3. The paper advances a color-based multivariate clustering analysis technique for filling image highlight that contributes to improve feature extraction precision of Cortical (C) and Posterior Subcapsular (P) Cataract images and conduces to enhance diagnosis and grading precision of C and P.

## 2 Materials and methods

**FIGURE 1:**
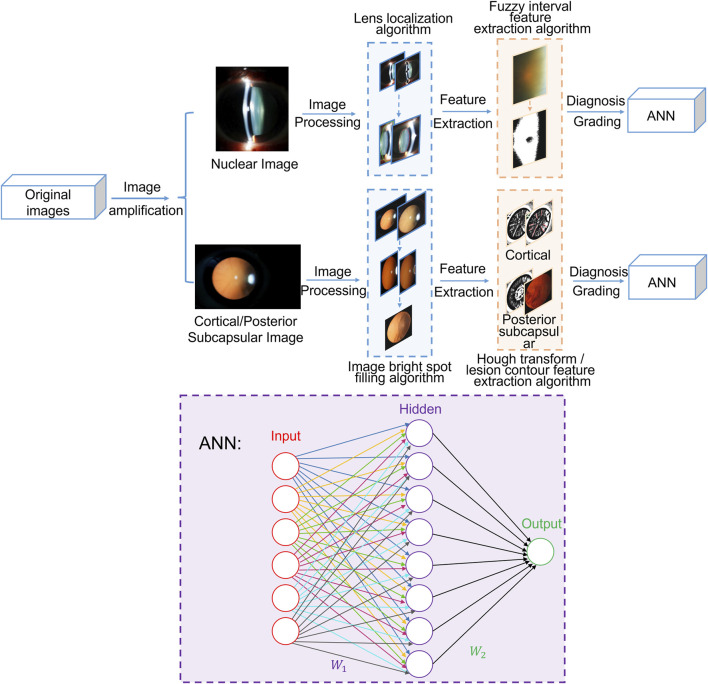
The overall diagnosis framework for cataract.

The experimental protocol was established Helsinki according to the ethical guidelines of the Declaration and was approved by the Ethics Committee of The Second Affiliated Hospital of Harbin Medical University. Written informed consent was obtained from all patients before collection. In this section, this study is expanded from two models: the nuclear cataract diagnosis and grading module and cortical and posterior subcapsular cataract diagnosis and grading module. As shown in Figure 1, the total algorithm framework includes preprocessing, feature extraction and cataract grading neural network and other processing. The artificial neural network (ANN) consists of two layers and employs the sigmoid function as its activation function.

For nuclear images, the preprocessing stage involves eliminating bright spots caused by flash light and employing the expanded ellipse traversal method to achieve lens localization. During feature extraction, the fuzzy interval scale method is adopted to obtain feature information regarding Nucleur Opalescence (NO) and Nuclear Color (NC). Finally, the extracted feature information is input into the ANN to enable the automated diagnosis and grading of nuclear cataracts.

For cortical and posterior subcapsular images, the preprocessing stage includes image segmentation using the minimum circumscribed circle method and filling image bright spots through color multivariate cluster analysis. In the subsequent feature extraction phase, the Hough transform is employed to detect line information in the images as feature information for cortical cataracts, and lesion contour information is extracted as feature information for posterior subcapsular cataracts. Ultimately, this extracted feature information is fed into the ANN to facilitate the diagnosis and grading of cortical and posterior subcapsular cataracts.

### 2.1 Dataset and statistical methods

**FIGURE 2:**
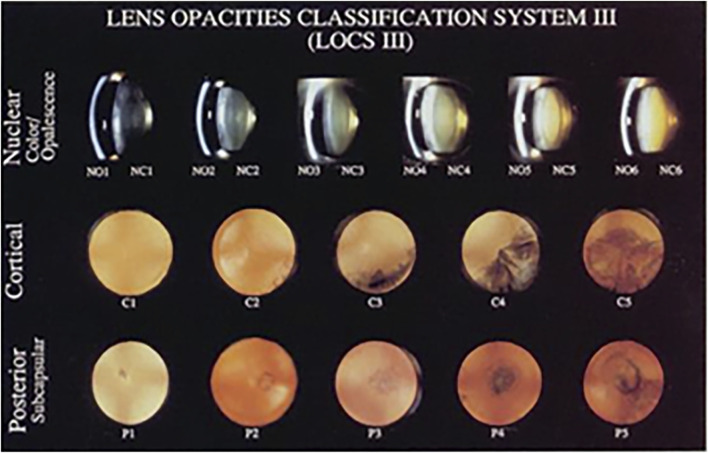
Lens opacities classification system Ⅲ, LOCS Ⅲ Chylack et al., 1989.

All slit-lamp photographs were obtained from the Department of Ophthalmology of the Second Affiliated Hospital of Harbin Medical University from 2019 to 2022, including 1,003 photographs of normal lenses and cataracts of different severities. Each photograph was taken under mydriatic conditions. Different modes were used: slit-beam mode was used for NO and NC evaluation, and retro-illuminated photographs were used to assess C and P based on LOCS III (Figure 2). Slit-beam photos were taken with an angle greater than 15° between the illumination arm and the viewing arm, while the retro-illuminated photographs were taken with a frontal view of the lens.

The exclusion criteria for the photo were: (1) pupil diameter ≤ 5 mm in mydriatic conditions or unclear image; (2) other special types of cataracts; (3) presence of other anterior segment diseases, trauma, surgical history and so on.

The data sets were partitioned. After that, each type of anterior segment images was divided into two disjoint subsets. The training set accounts for 70% of the data, while the test set accounts for 30% (Krizhevsky et al., 2017). The dataset comprised 1,003 anterior segment images, including 215 from healthy lenses and 788 from cataractous lenses classified per LOCS III criteria. All images were categorized by modality: slit-beam illumination (n = 717) and retro-illumination (n = 286). Healthy lenses were uniformly graded as NO0/NC0, while cataract severity followed LOCS III grading (NO1-6, NC1-6, C0-5, P0-5) (Supplementary Tables S1-S2).

To address the limited original image dataset, we need to augment it to prevent model overfitting and improve algorithm performance. Multiple methods were used to augment the data set, including adding salt and pepper noise, gaussian noise, dimming the image, brightening the image, rotating, mirror flipping, mix-up, and more.

### 2.2 Nuclear cataract diagnosis and grading module

#### 2.2.1 Preprocessing

##### 2.2.1.1 Removing bright spots

**FIGURE 3:**
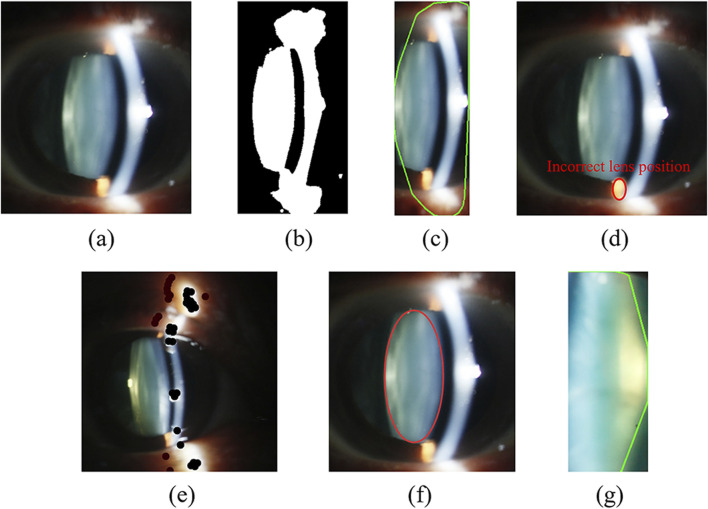
Nuclear image preprocessing under slit-beam photos. **(a)** Original image; **(b)** Binary result of Fig. **(a)** affected by highlighted noise; **(c)** Localization results of oversized lens; **(d)** Incorrect lens positioning results; **(e)** The result of drawing black dots; **(f)** Lens localization result based on expanded ellipse traversal; **(g)** Intercepted lens area.

The original image is obtained by using the slit-beam mode of the anterior segment. Due to the influence of flash light, there are usually two kinds of light spots in the nuclear type image: one is white light spots due to the reflection of cornea and the other is yellow spots on the skin near the eye, as shown in Figure 3a. Due to reflection of eyes and local uneven illumination, the quality of original images is impacted, which may hinder the detection and grading of cataract precisely.

Figure 3c is a lens contour obtained using a basic contour extraction algorithm for Figure 3b, which contains a large amount of interference information. The highlighted area in Figure 3d is the region where inaccurate localization of the lens occurs due to the influence of bright spots. Therefore, in this paper, we first use the method of drawing black dots on bright areas with three RGB values above 250 to reduce the high-brightness light spot, as shown in Figure 3e. In this way, it is impossible to form a large internal ellipse in the bright area and the contour area where the lens is located is maximized. Then, the algorithm converts the original image into a gray image, and uses dynamic threshold to binarize the gray image.

##### 2.2.1.2 Lens localization algorithm of nuclear image based on expanded ellipse traversal

Figure 3c illustrates the imprecise lens contour obtained using basic contour detection methods, which contains a lot of useless information, thereby compromising the accuracy of feature extraction. Therefore, prior to implementing the DL model, it is necessary to accurately extract the region of interest from the original image. This section proposes a lens localization method based on extended elliptical traversal for kernel images, which processes the binarized image in Figure 3b.

Before that, we need to use the contour search function to find the approximate position of the lens contour, and find the minimum rectangular boundary covering this contour to obtain the position coordinates, width and height of the upper left corner of the rectangle. We think that the lens contour circled by the contour search function is approximately the maximum contour. Next, we will use the approximate coordinates and other information to accurately locate the lens. During the positioning, we first traverse all rectangles in the maximum contour and obtain their inner ellipses. Then we need to traverse all points in the bounding rectangle to determine whether they are inside the ellipse. If so, we need to check whether the point is white. Once some spots inside the ellipses are not white, the ellipse does not meet the conditions and other ellipses need to be traversed. In fact, the ultimate goal of the algorithm is to find the largest elliptical area that is completely filled with white. Therefore, the algorithm eventually obtains the largest ellipse after many iterations, as shown in Figure 3f.

The specific implementation process of lens positioning algorithm is shown in Algorithm 1. The input of the algorithm is that we use the contour search function in computer vision to get the largest possible area of the lens, and obtain the coordinates (*x*_1_, *y*_1_) of the top left corner of the outer rectangle of the area, as well as the width *w*_1_ and height *h*_1_.

Algorithm 1 Eye lens location algorithm based on maximum ellipse search.

 **Input:** Binary image *src* to be detected (*x*_*1*_, *y*_*1*_), *w*_*1*_ and *h*_*1*_
 **Output:** Position coordinates (*x*_*2*_, *y*_*2*_), width *w*_*2*_ and height *h*_*2*_ of lens
 **Begin**
  Input binary image *src*;
  **While** The ellipse area that meets the following conditions is the largest:
   Traverse all rectangles *rect* within the width w_1_ and height h_1_ range;
   Obtain the inscribed ellipses *ellipse* of the rectangles *rect*;
   **If** all points within the *ellipse* are white:
    Calculate the elliptical area that meets the condition;
    Obtain the outer bounding rectangle corresponding to the ellipse with the maximum area;
    Output The coordinates of the top-left corner (*x*_*2*_, *y*_*2*_), width *w*_*2*_, and height *h*_*2*_ of the rectangle.
 **End**

The lens part is not strictly elliptical shape, resulting in the ellipse not containing the complete lens. Therefore, it is necessary to fine-tune the size of the ellipse to obtain the final lens positioning result. Finally, the algorithm intercepts the lens portion for subsequent feature extraction, as shown in Figure 3g.

#### 2.2.2 Nuclear cataract diagnosis and grading neural network

##### 2.2.2.1 Feature extraction

The diagnosis and classification of nuclear cataract include NO and NC. We make use of color proportion to classify, in which cyan pixels are used to judge NO and yellow pixels are used to judge NC. In this part, we use a kernel image color eigenvalue extraction algorithm based on fuzzy interval scale. The specific process is as follows.

We first need to set the standard color (cyan or yellow) and the offset interval *Offset*. We assume that the RGB of the standard color is (r,g,b). According to this, we can calculate the corresponding fuzzy interval, which are respectively R = [*r-Offset,r + Offset*], G = [*g-Offset,g + Offset*], B = [*b-Offset,b + Offset*]. It is not difficult to see that different standard colors and offset intervals will obtain different fuzzy intervals. After that we traverse all pixels in the image and count the proportion of pixels points whose RGB values are in the fuzzy interval, which is color feature of nuclear type image.

##### 2.2.2.2 Lens nucleus diagnosis and grading method based on neural network

The input of the neural network is the ratio value of cyan or yellow pixels in the lens image after image expansion, positioning and clipping. The input data set is divided into training set and test set according to the ratio of 7:3. The output layer of the neural network is a point, representing the grade of the nuclear classification. After the above processing, we have obtained a complete nuclear cataract diagnosis and classification model, including the classification of Nucleus Opacification and Nucleus Color.

### 2.3 Cortical and posterior subcapsular cataract diagnosis and grading module

#### 2.3.1 Preprocessing

Before feature extraction, we need to preprocess the original image, which mainly includes two steps: image positioning segmentation and bright spot filling. This phenomenon occurs because the eyeball’s three-dimensional structure generates luminance gradients under unidirectional illumination. At the same time, flash lights make bright spots unavoidable in the image. These problems will interfere with feature extraction, so preprocessing is needed.

##### 2.3.1.1 Image segmentation technology based on minimum circumscribed circle

**FIGURE 4:**
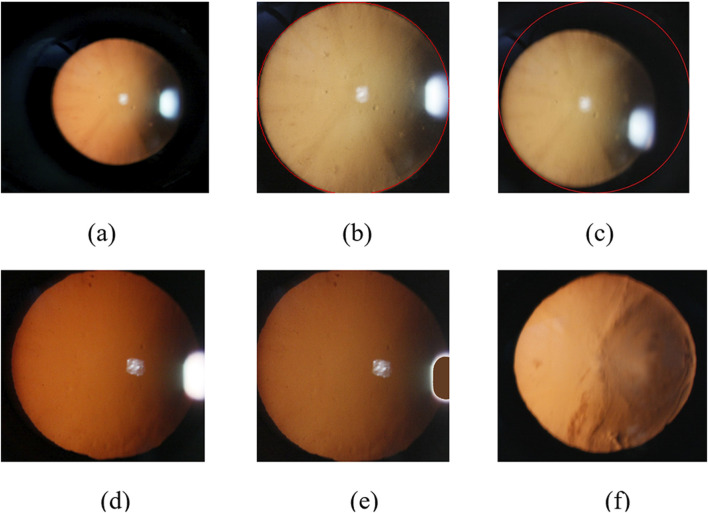
Slit-beam photos preprocessing. **(a)** Original image; **(b)** Ideal result using minimum circumscribed circle; **(c)** Inaccurate eye region positioning results; **(d)** Unfilled Image; **(e)** Filled image; **(f)** Filled color image after debugging.

The eyeball occupies only a small part of the original image. In order to obtain the features of the eyeball image, it is necessary to locate the eye part of the original red light reflection image. In this paper, we use the method of minimum circumcircle to segment the original images to remove the information of dark features. Figure 4b shows the ideal result of interception, which can accurately extract the eyeball region.

However, there are often several bright spots in the real cataract eyeball images due to the existence of flash during the photographing process, and the image often exist uneven light and dark distribution because of the three-dimensional shape of the eyeball, which will lead to inaccurate positioning results of some images using the above method, as shown in Figure 4c.

Therefore, in order to solve the above inaccurate results, we first use bright spot filling technology to remove noise, and then locate the part of the eyeball.

##### 2.3.1.2 Image bright spot filling technology based on color multivariate cluster analysis

Due to local uneven illumination and reflection of eyes, the quality of original images are impacted, which may hinder the detection and grading of cataract precisely. Therefore, we adopt color filling to make the RGB values approximately consistent between bright spot area and the surrounding pixels to eliminate the effect of bright spots.

We use the idea of averaging to select the required fill color. The algorithm first classifies the image pixels in the circular domain using the idea of clustering, which can be roughly divided into three categories: yellow, white and black. Then, the algorithm calculates the mean value of the RGB values of all pixels in the yellow classification. Finally, the algorithm fills the bright spot area with the color corresponding to the mean value, as shown in Figure 4e. The specific implementation process of the algorithm is as follows (Algorithm 2).

Algorithm 2 Image bright spot filling technology based on color multivariate cluster analysis.

 **Input:** the image *img* of preserving the circular target area of the eyeball
 **Output:** Color image *src* after removing bright spots
 **Begin**
  Input the image *img* with bright spots;
  An array B is defined to indicate whether clustering has been completed;
  Array C is defined to represent the categories of clustering;
  **Foreach** (all pixels in the image):
   Calculate the color space distance *distance* between any two pixel points;
   Save all *distance* information to the list *list*;
   Traverse *list* to obtain the minimum distance;
   Locate the two pixel points corresponding to the minimum distance in the image and calculate the average of the RGB values of the two points;
   Update the values of arrays B and C;
  Based on distance information, a simple clustering method is used to cluster all pixel points into three categories;
   Obtain the average value *Avg* of all pixels clustered into a yellow class;
   Traverse the pixel points in the image, assign the RGB value of Avg to the pixel points at the bright spot position, and obtain a new image *src.*
 **End**

After the above processing, we can restore the image color and retain the image information of the bright area of the image. The preprocessing results of cortical or posterior subcapsular images after multiple adjustments is shown in Figure 4f.

##### 2.3.1.3 Image circular contour detection technology based on Hough transform

In this section, we conduct more precise eyeball localization on the image after removing the bright spots. We first convert the colored eye image into a grayscale image, then set a suitable fixed threshold to remove some noise, and finally use Hough transform to detect the circular contour of the eyeball image.

#### 2.3.2 Cortical and posterior subcapsular cataract diagnosis and grading neural network

##### 2.3.2.1 Feature extraction

**FIGURE 5:**
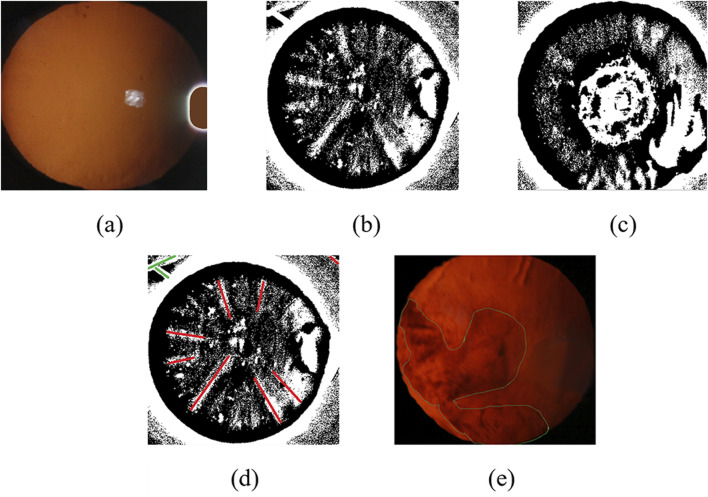
Slit-beam photos feature extraction results. **(a)** Filled color image after debugging; **(b)** Suspected cortical binarization image preserving lesion information; **(c)** Suspected posterior subcapsular binary image preserving lesion information; **(d)** Result of binary original image line fitting based on Hough transform method; **(e)** Original image contour extraction results.

In order to preserve the images feature of the lesion in the eyeball, we use an adaptive threshold method to process the images. We first convert the color image obtained after removing the bright spots into a grayscale image *Grey*, and then use a fixed threshold method to obtain a binary image with the largest circular contour, which contains complete lesion information. Finally, we adopt an adaptive binarization method to process the grayscale image *Grey* combining the above circular contour to obtain binarized images that preserve the lesion information, as shown in Figures 5b,c.

There are differences feature information in binary images of cortical and posterior subcapsular cataract. It is necessary to adopt other feature extraction methods to obtain more accurate feature information in binary images. We use Hough transform to detect line information in binary images, including the length, position, and distance between the line and the center of the circle, which are used as feature information of cortical cataract, as shown in Figure 5d. Subsequently, we extracted the lesion contour information from the binary image, including the area ratio, perimeter ratio, and contour centroid of the contour to the circular contour of the eyeball, which are used as feature information of posterior subcapsular cataract, as shown in Figure 5e.

##### 2.3.2.2 Cortical and posterior subcapsular cataract diagnosis and grading method based on neural network

The input of the neural network is the feature information obtained from the cortical and posterior subcapsular cataract images in the previous section. The input data set is divided into training set and test set according to the ratio of 7:3. The output layer of the neural network is a point, representing the classification of the cortical and posterior subcapsular cataract. After the above processing, we have obtained a complete cortical and posterior subcapsular cataract diagnosis and classification model.

## 3 Results and analysis

The diagnosis and grading results of cataract was evaluated using a confusion matrix. To comprehensively assess the diagnostic and grading performance of the neural network, the following metrics were employed: Precision, Recall, F1-score, Accuracy, Receiver Operating Characteristic (ROC) curves and the Area Under the Curve (AUC). Precision, recall, and F1-score were primarily utilized to evaluate the classification effectiveness of various categories within the system, while Accuracy and AUC were employed to assess the overall performance of system. A total of 1,003 original images were marked, with 715 slit-lamp images used to differentiate between NO and NC, and an additional 288 retro-illumination images used to differentiate between C and P. In this study, we set level zero, NO0 is the transparent lens nucleus, NC0 is the normal color of lens nuclear, C0 and P0 is the transparent area of lens cortex and posterior subcapsular.

**FIGURE 6:**
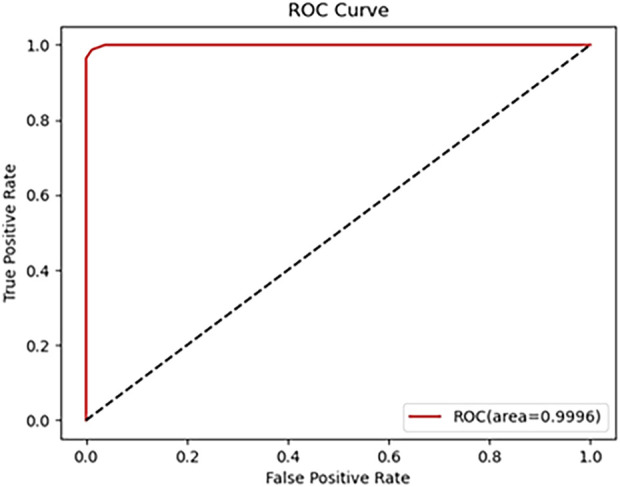
ROC Curve of the cataract grading system.

As shown in Table 1, the metrics for C and P outperform those for NO and NC. which can achieve 96.88% classification Precision, 98.41% F1-score and 98.26%Accuracy, with all other metrics at 100%. It can be seen that the overall accuracy of the Neural Network for diagnosing is above 92.28%, and the accuracy for C is the highest, which is 100%. The AUC is 99.96% (shown in Figure 6). These results indicate that the proposed algorithm performs well in the classification of various cataract types.

**TABLE 1:** Recall, Precision, F1-Score, AUC and Accuracy of different types of cataract.

Types	Precision (%)	Recall (%)	F1 (%)	AUC (%)	Accuracy (%)
NO	84.56	98.24	95.30	96.68	92.28
NC	99.48	96.45	97.94	99.55	97.19
C	100.00	100.00	100.00	100.00	100.00
P	96.88	100.00	98.41	100.00	98.26

### 3.1 Result of NO grade

**FIGURE 7:**
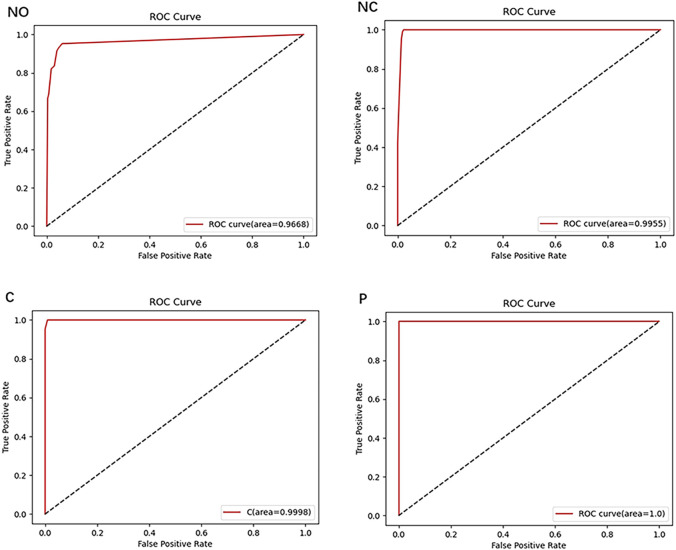
ROC Curves of the NO, NC, C, and P grading.

In this study, a neural network was utilized to categorize anterior segment images captured by slit-lamp photography into seven levels ranging from NO0/Normal to NO6. The overall accuracy exceeded 90.88%, with an AUC of 96.68% (shown in Figure 7). As illustrated in Table 2, concerning the NO classification outcomes of the neural network, the recall for NO1, NO2, and NO4 all exceeded 91.46%, indicating satisfactory performance of the system in these NO classifications. While the model demonstrated high sensitivity for early nuclear opacity (NO1, 95.24%), its performance was more limited for normal lenses (NO0, 68.97%). This suggests inherent challenges in detecting subtle features of normal lenses from static images compared to clinical dynamic evaluation.

**TABLE 2:** Recall, precision and F1-Score of NO, NC, C, and P.

Types	Classification	Recall (%)	Precision (%)	F1-score (%)	Accuracy (%)
NO	NO0/Normal	68.97	99.91	78.43	92.44
	NO1	95.24	62.50	75.47	95.44
	NO2	91.46	90.36	90.91	94.74
	NO3	83.58	78.87	81.16	90.88
	NO4	92.86	61.90	74.29	93.33
	NO5	82.14	92.00	86.79	97.54
	NO6	66.67	90.91	76.92	97.89
NC	NC0/Normal	98.86	92.55	95.60	97.19
	NC1	100.00	72.73	84.21	97.89
	NC2	98.61	95.90	97.26	98.60
	NC3	95.23	93.75	94.49	97.54
	NC4	100.00	61.54	76.19	98.25
	NC5	57.14	80.00	66.67	98.60
	NC6	41.94	100.00	59.09	93.68
C	C0/Normal	100.00	100.00	100.00	100.00
	C1	100.00	100.00	100.00	100.00
	C2	95.24	95.24	95.24	98.26
	C3	100.00	96.00	97.96	99.13
	C4	91.67	100.00	95.65	99.13
	C5	75.00	75.00	75.00	98.26
P	P0/Normal	96.23	100.00	98.08	100.00
	P1	100.00	100.00	100.00	98.26
	P2	100.00	86.67	92.86	98.26
	P3	100.00	60.00	75.00	98.26
	P4	100.00	100.00	100.00	100.00
	P5	91.30	100.00	95.45	98.26

### 3.2 Result of NC grade

As shown in Table 2, for the classification ranging from NC0 to NC6, the overall accuracy surpassed 93.68%, with an AUC reaching 99.55% (as illustrated in Figure 7). Specifically, the classification recall for NC0-NC4 all exceeded 95.23%. These results indicate excellent performance of the proposed neural network in the task of NC classification.

### 3.3 Result of C grade

The proposed algorithm subdivides anterior segment images under red reflex into levels ranging from C0/Normal to C5. The overall grading accuracy exceeds 98.26%, with an AUC value of 99.98%. As demonstrated in Table 2, the recall for levels C0 to C4 all remain above 91.67%, while precision exceeds 95.24%. This algorithm exhibits significant advantages in distinguishing non-severe cortical cataract.

### 3.4 Result of P grade

The overall accuracy for the six grades P0 to P5 reached 98.26%, according to Table 2, with an AUC of 100% (as illustrated in Figure 7). The recall in the P classification exceeded 91.30%. These results demonstrate a significant advantage of the algorithm in P classification.

## 4 Discussion

In this paper, we propose a novel paradigm for automatic cataract detection. This study first expanded the dataset and divided it in a 7:3 ratio. Subsequently, several image preprocessing methods were proposed, including a lens localization algorithm for nuclear cataract images and a bright spot filling algorithm for cortical and subcapsular cataract images (Son et al., 2022). The processed images were then diagnosed and graded using deep learning algorithms. In terms of diagnostic results, the neural network achieved accuracies of 92.28%, 97.19%, 100%, and 98.26% for the NO, NC, C, and P classifications. respectively, demonstrating good image recognition performance under retro-illumination conditions. Regarding grading, the algorithm achieved accuracies exceeding 92.28% for NO grading, over 97.19% for NC grading, and above 100%,98.26% for C, P grading, particularly excelling in C and P grading. To further contextualize our findings, we compare our results with recent state-of-the-art approaches in automatic cataract detection (Table 3). While previous studies predominantly focused on specific cataract types (e.g., Wu et al., 2019 on mixed cases or Shimizu et al., 2023 on nuclear cataracts), our method achieves both high accuracy (up to 100% for C grading) and broad generalization across NO, NC, C, and P categories. Our model consistently outperforms these benchmarks while maintaining strong sensitivity and specificity.

**TABLE 3:** Comparative performance analysis of AI models in automatic cataract detection.

Authors	Samples (images)	Typle of cataract	AI algorithms	AUC	Accuracy (%)	Sensitivity (%)	Specificity (%)
Wu et al. (2019)	37,638	normal lens, cataract or postoperative eye	ResNet	95.96%	88.79%	92.00%	83.85%
Wu et al. (2022)	16,200	cataract and noncataract	CNN	>91%	>84%	>71%	>89%
Acharya et al. (2010)	140	normal lens, cataract or postoperative eye	ANN	-	93.3%	98%	100%
Gan et al. (2023)	647	cortical cataract	FCNResnet50	>90%	-	-	-
Shimizu et al. (2023)	38,320	nuclear cataracts	Grad-CAM	93.4%	94.2%	96.2%	93.1%

After systematic analysis of diagnostic error causes, three primary biases were identified: Firstly, inconsistencies in exposure intensity impair feature extraction efficacy, particularly in underexposed regions. Secondly, unilateral illumination in slit-lamp systems induces image shadows that mimic pathological lesions, significantly complicating accurate lesion identification in shadowed areas. Thirdly, dataset limitations critically constrain artificial intelligence performance: recognition accuracy exhibits strong dependence on both training data volume and feature diversity, adhering to the scaling laws demonstrated in ophthalmic AI studies (Zhao et al., 2023).

To this day, Artificial intelligence algorithms have been applied to the diagnosis and grading of cataract in fundus images. Early on, Xu et al. employed a CNN model to analyze fundus images for cataract diagnosis and grading (Xu et al., 2020). In the early years, in clinical practice, ophthalmologists rely more on observing anterior segment images under a slit lamp for intuitive and accurate cataract diagnosis. Wu et al. utilized anterior segment images to develop a remote cataract screening platform based on deep learning algorithms, specifically targeting nuclear cataract (Wu et al., 2019). This study categorized cataract into mild, moderate, and severe, providing corresponding treatment recommendations. However, it is worth noting that this study focused solely on nuclear cataract and did not provide precise grading results, which somewhat limits the AI system’s ability to follow up and assess patients’ conditions. In contrast, our algorithm design offers several significant advantages: Firstly, we employ the more precise LOCS III criteria for cataract diagnosis and grading, ensuring diagnostic accuracy. Secondly, our study explicitly delineates the current severity of cataract, providing robust support for patient follow-up. Lastly, we not only investigate the diagnosis and grading of nuclear cataract but also encompass cortical and posterior subcapsular cataract, thus achieving a more comprehensive study for cataract.

This study explores the application of artificial intelligence in the diagnosis and grading of cataract. The application of this technology has the potential to significantly optimize healthcare delivery for remote areas, impoverished communities, and elderly patients, addressing challenges such as long-distance travel and high costs, thereby reducing the economic burden on the populace (Ting et al., 2019). For diagnosed cataract patients, the technology provides a relatively standardized severity index, facilitating follow-up visits and optimizing patients management. In clinical practice, the implementation of this system is expected to enhance the efficiency of healthcare providers, allowing ophthalmologists to serve more patients and increase screening rates. Furthermore, the objective data parameters provided by the system can offer standardized guidance for surgical operators, thereby enhancing surgical safety. In conclusion, this study provides novel insights for future research and underscores the significance of integrating emerging artificial intelligence technologies into clinical practice. During the image collection process, we encountered several challenges. (1) The eyeball is a three-dimensional structure, while anterior segment images are two-dimensional. When attempting to focus on a specific point, surrounding features may appear blurred to varying degrees. Therefore, during image acquisition, multiple adjustments of focus were necessary to capture images at different planes, demanding precise alignment of the focus on specific points on the lens in clinical practice. (2) The majority of patients in the clinic present with various types of cataracts. When patients have two or more types of cataracts, the images obtained in practice are often not as clear and discernible as those displayed in the LOCS III. (3) In clinical practice, there is significant variability in the number of images collected for each grading, and the study lacks sufficient data support.

## 5 Conclusion

This study has successfully developed a novel artificial intelligence system for identifying nuclear cataract, cortical and posterior subcapsular cataract. Among the various types of cataracts, the system demonstrates particularly outstanding accuracy in identifying cortical cataract. Not only does this neural network system possess the capability to diagnose the types of cataracts, but it also accurately grades the severity of cataract into NO, NC, C, and P. Particularly noteworthy is its performance in grading between C and P, where the system excels. Furthermore, the system exhibits superior sensitivity in cataract grading when the opacity of the lens is at a lower level compared to higher opacity levels. These findings underscore the promising prospects of artificial intelligence in cataract diagnosis and grading. Hence, continued efforts should be directed towards the development and optimization of more precise algorithms to facilitate its widespread application in clinical practice.

## Data Availability

The original contributions presented in the study are included in the article/Supplementary Material, further inquiries can be directed to the corresponding authors.
